# The short-term results of an MI-adapted and culturally tailored intervention on self-reported oral health behaviors of indigenous caregivers and their children

**DOI:** 10.3389/froh.2025.1620597

**Published:** 2025-09-30

**Authors:** Christine Kirby, Julie Baldwin, Kristan Elwell

**Affiliations:** 1 Center for Community Health Engaged Research, Northern Arizona University, Flagstaff, AZ, United States; 2 Health Sciences Department, Northern Arizona University, Flagstaff, AZ, United States

**Keywords:** indigenous oral health, cultural tailoring, social determinants of health, oral health intervention, oral health

## Abstract

**Introduction:**

American Indian (AI) children have the highest incidence of dental caries of any ethnic group with 4 times the cases of untreated dental caries compared to white children. Great Beginnings for Healthy Native Smiles (NIDCR U01DE028508), a community focused oral health intervention, included culturally tailored oral health education materials utilized alongside adapted motivational interviewing (MI) techniques to promote oral health care and education at home.

**Methods:**

The intervention was conducted by local community members from two partnered Indigenous communities. Using formative assessment data from semi-structured caregiver and provider interviews, session transcript data, and debriefing interview data from participants post-intervention, the objective of this study was to determine the impact of culturally tailored oral health education focused on behavior change in each community.

**Results:**

Results suggest that discussion with community health representatives using adapted MI and culturally tailored materials can result in self-reported behavioral change.

**Discussion:**

The findings highlight the importance of involving trusted community health workers in delivering culturally tailored oral health messages for mothers and their children to reduce ECC.

## Introduction

American Indian children have the highest incidence of dental caries of any ethnic group, with 4 times the cases of untreated dental caries compared to white children (1). The Great Beginnings for Healthy Native Smiles (GBHNS) intervention was designed and implemented over a 6-year period, working closely with two Indigenous communities (one Plains tribe and one Southwestern tribe). Using data from a formative assessment (2), quarterly feedback from Community Advisory Boards, and a small pilot study, the GBHNS team designed 6 oral health focused sessions for pregnant women or new caregivers applicable for mothers from pregnancy to the child's age of 3 (Baldwin, et al., under review; Kirby, et al., under review). Each session was focused on milestones for the child's relevant age or on prenatal oral health for expecting mothers. Both sites customized the language, examples, imagery, and overall feel of each session. Incorporated into each session script were values and practices central to Motivational Interviewing (MI) (3–5).

Using a social determinants of health (SDOH) framework to better situate the oral health education for each community, the team culturally tailored all educational materials for each site (6). The World Health Organization defines SDOH as “the conditions in which people are born, grow, work, live, and age, and the wider set of forces and systems shaping the conditions of daily life” (7). By identifying local barriers and supports to oral health care in each community in the formative assessment, GBHNS was able to design culturally tailored oral health materials focused on troubleshooting typical oral health issues families in the two tribes may experience while also connecting families with local resources (programs such as WIC, Head Start, farmer's markets, local healthy foods etc.). Previous studies focused on Indigenous oral health education have utilized motivational interviewing and culturally tailored materials (8–11). These studies focused on building culturally tailored oral health education programs to impact oral health behaviors (and ECC) in Indigenous populations. GBHNS built upon the successes of these studies, responding to SDOH from the formative assessment (e.g., access to oral health care, including transportation), and the use of MI.

Public health and human behavior research has noted a need for more research to provide a deeper understanding of relationships and interactions between health providers and their clients (12). Research suggests that when nurses and other community health providers are trusted, they can implement interventions which could change health behaviors for the better (12, 13). Additionally, within medical anthropology and public health, Community Health Workers (CHWs) are viewed as brokers between the health care systems and the communities in which they work (14). In the partnered Indigenous communities, instead of the title “CHW,” the community staff are referred to as Community Health Representatives (CHRs). Individuals who were hired and trained as CHRs in this project not only helped with the adaptation of the intervention (brokering cultural information to the research team to better serve the community), but they also brought oral health information they found to be missing back to their communities. Becoming a bridge between providers and their community members, GBHNS CHRs were recruited to assist with adapting the oral health education to fit their community and bring evidence based oral health care education to new mothers and caregivers.

The objective of this paper was to explore the impact of a culturally adapted oral health education intervention utilizing adapted MI with two Indigenous populations and provide examples of communication displaying “trust” in CHRs in this specific context. While “trust” was not a measure in this specific study, the outcomes lend communication examples to be considered across dental health and community health settings. Trust in the case of this manuscript is expressed through a willingness for mothers and caregivers (to talk) openly about their family's oral health knowledge, practices, and beliefs. Through an analysis of formative assessment interview data, debriefing interviews, and intervention transcripts, we illustrate the importance of communication and trust in the oral health education environment. First, we illustrate a need for culturally tailored oral health education by revisiting data from the formative assessment (2). Then we focus on the adapted oral health education session transcripts implemented by community members (CHRs), and the self-reported behavior changes from study participants to explore the ways in which trusting relationships impact oral health education and at-home oral health behaviors.

## Setting

GBHNS partnered with two rural, Indigenous communities. One community is a Northern Plains Tribe and the other a Southwest Tribe. The two tribes share some characteristics: they are relatively smaller tribes (less than 15,000 members) located in rural regions of the United States, with a high prevalence of ECC, and limited oral health resources. The Northern Plains Tribe has an Indian Health Services (IHS) hospital and clinic, a Bureau of Indian Affairs (BIA), and the tribal government and programs. While all enrolled tribal members are eligible to seek dental and medical services at the IHS facilities on the tribal nation, those who live off the tribal nation often must travel a long distance (at least one hour away) for medical and social services. During the time of the intervention, the Northern Plains community had a federally funded Nutrition Program for Women, Infants, and Children (WIC) that helped families with healthy food. Their tribal Head Start Program had lost funding and was not functioning. The IHS dental clinic consisted of one head dentist, two general dentists, one pediatric dentist, one endodontist, one hygienist, and seven dental assistants.

The Southwest Tribe is also very rural and people who live on the reservation often need to travel 30–40 min for oral health care at the IHS clinic on the reservation. If in need of specialty care, they may have to travel as far as 150 miles. At the time of the intervention, seven dentists were stationed at the reservation healthcare center. The tribe's WIC program also assisted families with healthy food. Unlike the Plains Tribe, the Head Start program was functioning during the time of the intervention, assisting 200 primarily low-income children ages 3–5 with access to education and services.

## Methods

### Project overview

Beginning with a formative assessment with the partnered tribes, GBHNS recruited 57 oral health providers, medical providers, and program leaders of related concerns along with caregivers to discuss barriers and supports to oral health care for each population. Using this data, GBHNS’ formative assessment outcomes illustrated 3 main themes concerning the providers and caregivers: oral health knowledge and values, barriers to children's oral health, and supports for children's oral health (2).

In its next phase, GBHNS utilized the information from the formative assessment to collaborate with each community to culturally tailor each oral health education flipchart for each tribe (Kirby et al., in review). Community Advisory Boards (CABs), CHRs, and a small pilot informed the creation of the intervention sessions and adapted MI scripts.

We then conducted a feasibility study to determine cultural relevance and acceptability of the MI approach and the short-term impact of our oral health intervention sessions on the oral health knowledge, attitudes, beliefs, self-efficacy, and behavioral intentions of new mothers and caregivers. Various studies have measured the impact of MI on oral health with mixed results (9, 15–23) This variability may reflect the acceptability of MI in AI populations. Using culturally adapted materials alongside MI, we intended to study the acceptability of MI and whether pregnant women and/or mothers were ready to change their oral health behaviors (including changing behaviors related to their child's oral health). Our goal was to enroll 5 participants for each of the 6 oral health sessions (*n* = 30); however, we over-enrolled and achieved a sample size of 41 participants. Once participants received the session and debriefing interview, we conducted an analysis of the acceptability of adapted MI and culturally tailored oral health education. We asked questions about appeal, relevance, understandability, and acceptability of the approach and content of each session. We also assessed the pre-post session change in mothers' knowledge, attitudes, beliefs, confidence, and behavioral intentions as a supplementary line of inquiry. Results from the pre- and post-survey can be explored more in a forthcoming article (Baldwin et al., under review).

The adapted intervention launched in 2022–2023 and a total of 41 participants were recruited at both sites. Participants were recruited locally, by CHRs at health fairs, local maternal/child health programs, and by word of mouth. The intervention consisted of completing one culturally tailored oral health session with a CHR utilizing adapted MI, a pre and post survey, and concluded two weeks later with a debriefing interview over the phone.

### Formative assessment

The formative assessment data were the result of 57 in-depth semi-structured interviews at both sites with oral health providers, medical providers, program leaders (i.e., WIC, Child Protective Services) (*n* = 30) and those who identified as caregivers of children (*n* = 27). Snowball sampling was utilized to access providers who contributed to local oral health education or maternal/child health. Snowball sampling was also utilized with caregivers, identifying other caregivers within the community. Interviews were semi-structured to allow for more flexibility and constant comparative methods (24). Recruitment continued until thematic saturation was reached. Thematic saturation is reached once participants are no longer providing new or additional lines of inquiry on specific interview questions or prompts (24). Transcripts of interviews were coded using thematic analysis techniques and a constant comparative analysis in Nvivo (25). Nvivo is an analytic qualitative software program used for the storage, organization, and analysis of qualitative data. More can be read about the specifics of the assessment and outcomes in another publication of this project [see (2)].

### Intervention

After utilizing the formative assessment data and community input to culturally tailor the oral health education for both sites, 6 oral health education sessions were finalized at each site (Kirby et al., under review). The 6 sessions covered the following topics: maternal oral health, taking your child to the dentist, cleaning infant's mouth/brush child's teeth two times a day, limiting sweets/offer non-sugar foods and drinks, offering only plain water in bottles or sippy cups in bed, and reducing the sharing of objects that might spread germs. In addition to culturally tailoring the oral health education materials, CHRs and Community Advisory Boards also adapted MI to better fit each community. A “script” for each oral health session was created with an overall guide for what to accomplish with the participant and it included adapted MI prompts, with quick reference MI values and skills (i.e., “Reflect participant change plan”). CHRs were trained bi-weekly in MI. Training consisted of two hourly meetings each week with MITI assessments, practice sessions, and at home recordings with family members. In addition, two multi-day in-person training sessions were conducted at Northern Arizona University. The 6 oral health education intervention sessions were implemented at both sites in 2022–2023 as a feasibility study.

Eligibility criteria for mothers and caregivers included: (a) over 18 years of age; (b) currently pregnant and preferably in at least month 4 of pregnancy at enrollment; or with a child under 3 years of age. Mothers/caregivers were excluded from the study if: (a) they were not pregnant; (b) not American Indian or not giving birth to an American Indian child; (c) under the age of 18; (d) not able to understand or sign a consent form for themselves and their child to participate. All eligibility criteria were assessed in the REDCap mobile app (26). If eligible, those participants who consented to an intervention session also completed a pre- and post-intervention survey in REDCap.

A total of 41 eligible participants were enrolled. GBHNS conducted 27 sessions at the Northern Plains site and 14 sessions at the Southwest site. CHRs met participants at a location of the participant's choosing to conduct the intervention. For completing a session, participants were given a $50 gift card. Thirty-eight recordings of sessions were collected and transcribed using Trint.com and research staff review. Three sessions had damaged audio files or audio complications.

All recorded intervention sessions were thematically coded using ATLAS.ti, an analytic qualitative software program used for the storage, organization, and analysis of qualitative data (27). Intervention sessions were coded using structured coding techniques with predetermined definitions from the MITI 4.0 (i.e., cultivating change talk, softening sustain talk, reflection, question, affirmation, giving information, empathy). Deductively, themes were generated based on the literature, debriefing interview questions, and phenomena of interest. Inductively, themes were generated based on a grounded theory approach identifying and using participant's own words and experiences (24).

### Debriefing interviews

Two weeks post-intervention session, NAU Research Staff called each participant to conduct a recorded semi-structured debriefing interview to assess the appropriateness of the intervention session in addition to questions about the impact of the education session and any at-home oral health behavior changes. Semi-structured interviews were utilized for greater flexibility over the phone. For completing this session, participants were emailed a $25 gift card. If the participant did not have email, a physical gift card was issued to the local CHR and delivered to the participant. A total of 25 debriefing interviews were conducted, recorded, and transcribed; 12 participants were lost to follow-up, and 2 audio recordings were damaged. The debriefing interviewer asked participants to describe their knowledge of the CHR prior to the education session, how the session felt overall, if they found the information appropriate, if the session had an impact on them, if there was anything they would change about the session, and finally, if the session motivated them to change any at-home oral health behaviors.

All recorded intervention sessions and debriefing interviews were thematically coded using ATLAS.ti, an analytic qualitative software program used for the storage, organization, and analysis of qualitative data (27). Coding is a technique which allows researchers to turn qualitative data into nominal, ordinal, and interval-ratio variables for analysis (24). Debriefing sessions (*n* = 23) were coded deductively, themes were generated based on the literature, research questions, and phenomenon of interest. Inductively, themes were generated based on a grounded theory approach identifying and using participant's own words and experiences (24).

#### IRB

The Northern Arizona University Institutional Review Board approved this study on December 21, 2022 (IRB #1920796-6) with expedited review and the study was categorized as low risk; the study was also approved by the appropriate research review bodies of each Tribe. Informed consent was obtained for all interview participants, CAB members, and pilot participants at both sites. Data returned to each site will be returned according to each Tribe's data use agreement.

## Results

### Identifying a gap in oral health education

In the formative assessment interviews, themes of education and poor communication were frequent. Caregivers (*n* = 27) discussed a lack of understanding regarding available services, receiving oral health education from medical (not dental) providers, and overall negative experiences with oral health providers and services. Overall, these reports illustrated poor oral health communication and education at the community level.

Navigating changing resources can be confusing. Both caregivers and providers acknowledged the lack of specialty care locally and the coming and going of locally funded oral health education programs like Head Start. Caregivers voiced frustration with local oral health providers not explaining available services or a lack of specialty services. One caregiver reported:

My 2-year-old started getting cavities, so I would just constantly keep brushing them. It was tough because I had brought her to the local dentist here, but you know, because they don't have a pediatrician no more, they can't do nothing for her. So now her teeth are rotten all up on top. Her cavities are bigger than what they were when I took her in and they told me that, “Oh, we'll do a referral to whatever and they probably won't see her until she’s three.” I'm like, “What are you going to do for her?” [CH1]

Even in smaller communities, patients may not know or understand the exact services available to them at their local provider's office. In this case, the facility no longer had a pediatric dentist and was referring patients to other facilities about 2 h away. Even with this referral, the caregiver was unclear on when her child could be seen.

Of all 27 caregivers interviewed, 3 learned about oral health at the dentist office. The other 24 caregivers learned about oral health from local programming (i.e., WIC, Community Health Representative Programs), family members, or other parents. Family knowledge, or generational knowledge, was found to be a major support to children's oral health at both sites (2). Other work in global public health has shown that in rural communities, mothers often refer to more traditional systems of care rather than turning to formal medicine which appears true for GBHNS mothers and caregivers as well (12).

A majority of caregivers reported negative dental experiences from childhood. Some caregivers used the dentist as a scare tactic for kids in their family, “*You better brush or you’re going to have to go see the scary dentist!”* Other caregivers voiced visits filled with fear, “*Horrible memories… They didn't really talk to you about anything that was happening.” [CC10]*. Many of these negative experiences focused on dental procedures without localized anesthetic or procedures where children were restrained. Other experiences focused on the uncertainty or lack of communication and guidance from the dental providers.

Overall, caregivers did not view providers as a central source for oral health information. Because providers did not provide dependable services or clear communication according to caregivers, caregivers turned to other health providers and family members for oral health information.

On the other side, providers (*n* = 30) discussed failed attempts at communication and education with local patients, dropped programming, and the need for cultural experts to help reach at-risk patients. While oral health providers wanted to reach their patients and improve the oral health status of children at both sites, one provider lamented, “*After being here 11 years, I'd like to think I made a difference, and after looking back and seeing all the work we do, I don't know if we have, so it's very disappointing [18PH].”* Even adhering to best practices, providing fluoride varnish to children and regular visits, providers felt they were not reducing the severity of early childhood carries (ECC) in both populations.

Providers also shared experiences of breakdowns in communication with patients by describing their failed education attempts. While local school programming like Head Start was viewed as helpful, local oral health providers believed they need to do more educating within the community. One dental provider discussed a radio program where the head dentist would host an oral health show. The current provider explained why the program ended:

I don't think he wants to get back on there [radio station] because he did it for a few months. But it became overwhelming I think, work wise, going to the radio station and sitting there and hosting a show. But I think people need to know more about dentistry. I mean it's simple. Because I don't think anyone else is really teaching it. [19PH]

Though caregivers in the formative assessment expressed other avenues to accessing oral health information, there was a gap in oral health education at both sites and the theme of inconsistent funding for programming or abandoned education programs made it difficult for parents to know what and how to access resources.

Further complicating the oral health education gap, oral health providers described uninterested or absent parents in the clinic. Some of this could stem from a lack of cultural competency, though the communication is so poor it is difficult to tease apart (28). When asked if parents seem concerned to learn of decay in their child's teeth, a provider responded:

I don't think necessarily they're upset. No. Maybe concerned but that concern falls off as soon as they walk out the front door. Or if they have a notion they don't make it a point that you can tell.. It doesn't help now that in our clinic situation we don't have a pediatric dentist any longer. You know we've been missing one for a year and a half. [19PH]

Not knowing how to navigate conversations with parents at both sites was a consistent theme in the formative assessment provider interviews. Lacking services for children also caused confusion at one site. Without training to communicate with parents and their children, other providers were left to do what they could without specialized communication training. This left both patients and providers confused about how oral health information was received during visits.

Oral health providers often made assumptions about parent responses or resorted to communicating in ways they believed showed understanding. The provider below described how he communicates with local patients:

I make them look at me and listen to me. They have to look at me and listen to me. I don't know if they're used to that, but that's the only way I know they're listening to me. [18PH]

These uncertainties about communication were clear to some providers, and as a result, these providers wanted programming specifically to assist with oral health education for the community:

What I'm hoping is that if we have a culturally sensitive person involved in the field, maybe they [patients] get something different than what they get from me. Because when I talk to a patient, although I think I'm talking at their level and I try my best to not use medical terms or medical technologies that are, you know? You know how that is. We love using those words, but we try to talk with them at a level that they understand. And yet, when they get to the front desk, it's like I didn't even talk with them about something like that. [20PH]

Another provider shared the desire to have someone from the local Indigenous community to assist with at-home education promoting oral health behavior change:

So what I would like to see is when I'm treating a family that has high carries, that somebody .. I don't feel like I'm very good at changing people's behavior to do stuff. So it would be nice to have maybe a [Native] person that would go to their home or follow up with these high risk patients. [20PC]

One provider at a partnered location is a registered member of the local Tribe and she described her experience with local patients quite differently from other providers:

…at the same time, I'm very close to my patients. My patients trust me, utterly. They just really, we have such a good bond, that I don't see other patients having with the non-Native dentists. You know what I mean?

[18PC]

As a member of the local Tribe, this provider described an unspoken bond with their Indigenous patients and acknowledged how different it is for non-Indigenous providers.

The themes that emerged through the formative assessment interviews included a lack of oral health education, poor communication, and mistrust of oral health providers. These themes encouraged GBHNS staff to spend ample time tailoring the education materials, training local CHR in motivational interviewing techniques, and identifying local support systems participants could seek out if necessary. Creating embedded rapport building into the intervention through the use of adapted MI, GBHNS was designed to directly respond to community needs as expressed by both providers and caregivers.

### Intervention participants

We sought to evaluate the short-term impact of the culturally tailored oral health intervention sessions on oral health knowledge, attitudes, beliefs, self-efficacy, and behavioral intentions of new mothers and caregivers. The outcomes of the pre- and post-survey are explained more in the forthcoming Baldwin et al. 2025 publication. Sociodemographic characteristics for all participants were collected after consent in REDCap and are presented in Table 1.

**Table 1:** Caregiver sociodemographic and health characteristics (*N* = 41).

Characteristic	*N*
Age y, mean (SD)	27.4
Female gender	41
Relationship to child	
Mother	41
Hispanic ethnicity	
No	40
Yes	1
Race^a^	
American Indian/Native American^b^	40
Black/African American	1
White	1
Other	2
Education level	
<High school	6
High school diploma	18
Some college	13
College graduate or higher	4
Marital status	
Married/cohabiting	5
Divorced	2
Separated	2
Never married	11
Member of an unmarried couple	16
Other	5
Work force status	
Employed	19
Unemployed	9
Homemaker	3
Student	7
Other	3
Importance of maintaining your tribal identity	
Not at all	0
A little	1
Somewhat	9
Very much	31
Tribal language fluency	
I don’t speak my tribal language	10
I speak it a little, but not very well	26
I speak it moderately well	4
I speak my tribal language very well	1
Income adequacy	
Not enough to get by	4
Barely enough to get by	16
Sufficient to meet your needs	20
More than enough to meet your needs	1

^a^Responses may include more than one category.

^b^One participant did not endorse American Indian but did endorse membership in an eligible tribe.

### Increasing education: participant satisfaction and self-reported oral health behavior change

Participant feedback in debriefing interviews showed overall satisfaction with the sessions. Ten participants reported knowing the CHR guiding the session. Further, 16 participants indicated the sessions worked with their schedules since CHR were able to meet with them at a time and place of their choosing. Only 2 participants reported an issue in scheduling their session (both reported fussy babies as the reason for inconvenience). When asked if anything could have made the session better for them, 13 participants stated the session was “good as it was” and did not report any criticism. Participants did share their thoughts on improving the sessions. Six participants made 5 recommendations to improve the oral health education sessions including: more props for demonstrations, dedicated meeting space, shorter sessions, additional sessions, and a request for family education (not solely focused on the mother). Select excerpts from debriefing interviews regarding overall satisfaction with the culturally tailored oral health session are available in Table 2.

**Table 2:** Participant excerpt from debriefing interview.

Participant ID	Participant response to “Could the session have been better for you in any way?”
136-3	‪*It really, its like pretty. You gave a lot of information, for like, the way she gave me the presentation, I want to say, to where I could understand.*
137-2	‪*I think it was made good, like I mean, she explained everything articulately, particularly. I can't say it, she explained everything very well to me.*
138-9	‪*The way she did it, it seemed like she was covering all the points that she wanted to cover. She was taking that information and then remembering that that was part of the flipchart and she would even like flip ahead or flip back and be like, “Oh yeah.” And she kind of like, showed me the pictures. And I had shared with me a couple more pieces of information and then asked me if I was aware of or not.*
135-9	‪*I think it was, well laid out. … And it was informative that she gave me a lot of, um, a lot of information that I never had before.*
137-5	‪*I think the experience was pretty as good as it could get.*

Participants were asked if the session had any impact on them after the session had taken place. Participants then discussed the education they received and some even discussed information they had not known prior to their GBHNS oral health session. When asked if the information in the session motivated them to make changes in their at-home oral health practices, participants described the oral health behaviors they had changed at home. Of the 23 recorded and transcribed debriefing interviews, participants discussed a total of 10 different types of at-home self-reported behavior changes with an overall total of 27 reported behavior changes (Table 3).

**Table 3:** Debriefing interview: self-reported oral health behavior changes.

Debriefing self-reported changes	Total references 27
Brushing 2×/day	15
Cut down on soda/sweets	8
Improve diet	5
Going to/making dental appointments	3
Watching kids brush	3
Floss more	2
Modeling	2
Mindful of germs/kissing	1
None	1
Quit sippy cup	1
Rinse mouth after sweets	1

When comparing the goals participants set with CHRs at the end of an education session to the participant self-reported behavior changes in the debriefing interviews, 15 out of 20 were consistent. Of the total 27 debriefing interviews, only 1 participant reported not making any changes at home because of the program. During the intervention session, this participant made a goal of scheduling a dental appointment:

‪ **NAU Research Staff** [00:02:44] Well, as a result of participating in the session, is there anything that you might do differently for your oral health or for your child's oral health?

**137-4** [00:02:52] No, not really, really kind of impossible to get more into their dental appointments than being missed. [You either make the appointment or you do not.]

**NAU Research Staff** [00:03:06] That makes sense. And since participating in this session, has any of the information that CHR shared with you changed your oral habits or practices?

**137-4** [00:03:19] No not really. We still go about the same routine.

While 137-4 discussed getting into dental appointments for her children, she acknowledged that once these appointments are missed, they are missed; there is not much one can do to change that outcome. Accessing services frequently came up as an issue for caregivers in the formative assessment (*n* = 13), and perhaps making a goal of “making a dental appointment” was an easy goal to fail with the known local barriers. In the end, it is unlikely a patient would have been able to both schedule and attend a pediatric dental appointment in the two weeks following the oral health education session. Overall, 22 participants reported at least one changed behavior at home two weeks after participating in one culturally tailored oral health session delivered by a local CHR utilizing adapted MI.

### Building trust and increasing education with an adapted MI intervention

Each of the 6 culturally tailored oral health education sessions followed a basic format with built-in MI values: building rapport, identifying a session of interest to the participant, offering choice of where to start the session, culturally tailored and adapted MI educational session, setting a goal, and conclusion of session. Allowing participants to navigate the session and their interest, supports the patient-centered approach of MI. Building rapport, listening and responding to participant questions throughout the session allowed for a more successful goal setting discussion near the conclusion of the meeting.

Focusing on the goal-setting discussion in the oral health session, enabled us to investigate where the culturally tailored oral health education, adapted MI, and trust with the CHR came together in the intervention. Since this portion of the session involved going through participants' everyday routines at home, participants and CHRs often shared more personal details during this section. Table 4 contains the structure of the goal setting portion of the session which was adapted by CABs to better communicate oral health goals.

**Table 4:** Session guide: goal setting (change plan worksheet).

Change plan worksheet
1. “I realize we talked about a lot of different ways you can support your baby's oral health. Out of all the things we discussed today, is there one small change that feels doable right now to get started?”
2. “What led you to select that change?” (reflect change talk)
3. “What is it about that change that feels important to you?” (reflect change talk)
4. “Are there any other benefits you can think of to making this change?” (reflect change talk)
5. Flesh out details on goal if specifics have not been decided.
6. Confidence ruler question. Follow prompts.
7. Explore possible barriers.
8. Summarizing with the Change Plan

CHRs worked through an oral health behavior change goal, selected by the participant, for each session. Creating a plan to help participants lean on their own support systems during these types of barriers, allowed participants to talk through their ability to make changes in their oral health behaviors at home.

Demonstrating the ability to connect with participants and work toward creating an oral health behavior goal, CHRs were able to reflect on participants' experiences and struggles. Personalizing the conversation to fit exactly what the participant had shared with them throughout the session, CHRs were able to reach a level of engagement that may not be possible in a dental office and thus, allow for space to generate trust in oral health conversations.

**Figure 1:**
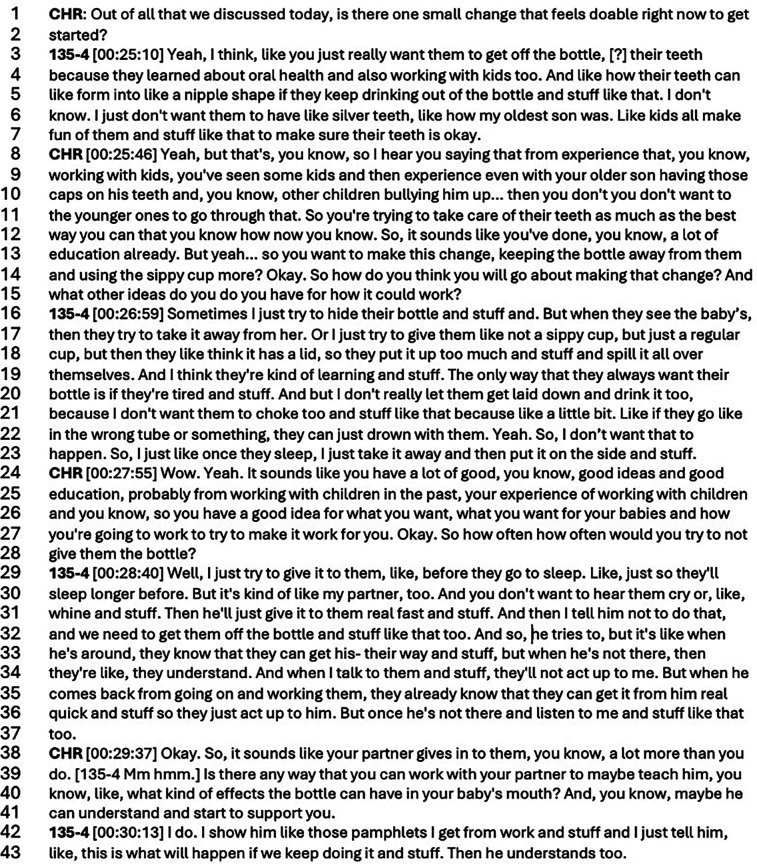
Transcript.

While dental providers described failed education attempts, confusing communication experiences, and an uncertainty about reaching patients, CHRs had remarkably different interactions with participants. Vulnerable and open conversations were observed during the goal setting portion of the oral health education intervention. Through the following excerpts (Transcript 1 and 2), adapted MI assisted in the flow of conversation between the participant and the CHR. The frequent questions, reflections, acknowledgment of expertise of participants(?), encouragement of participant decision making and autonomy, and building confidence were ways in which the CHRs were able to build trust with participants while leading them to incorporate oral health education into their daily lives. CHRs were able to “step into” participant lives, as a member of the community, and work together toward an individualized change-plan (Figure 1).

**Figure 2:**
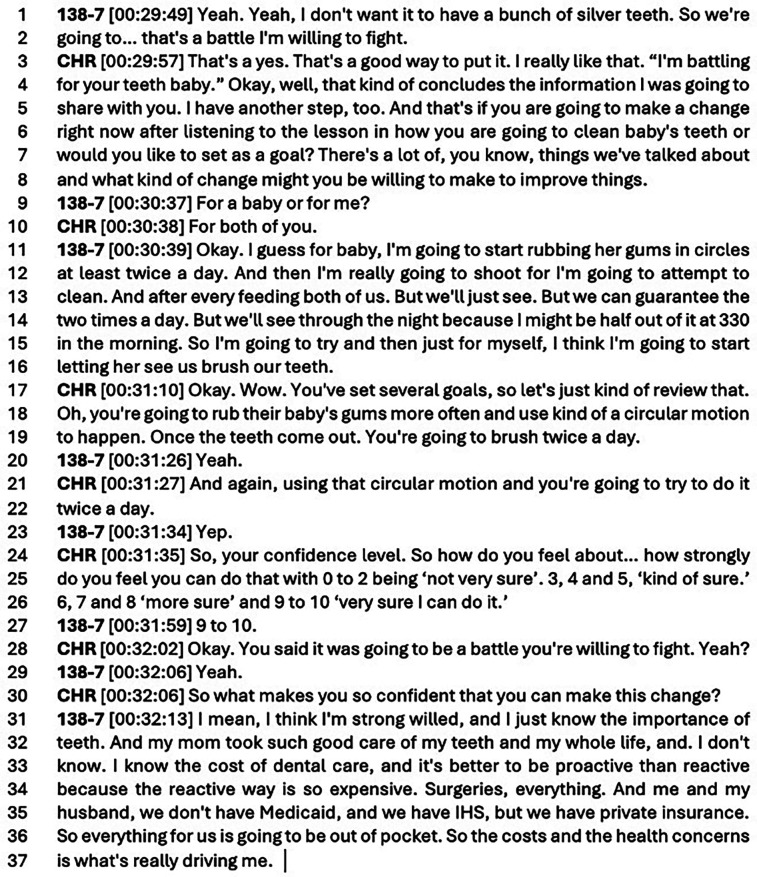
Transcript 2.

In the above excerpt, the participant discloses concerns around practices at home regarding getting her kids off the bottle (lines 3–8). We see the CHR encouraging the participant by discussing her experiences and valuing them as personal expertise (lines 29–33). Discussing interpersonal relationships at home (lines 36–42) with others can be difficult but here we see the outcome of a trusting relationship between the CHR and the participant. This trust is also illustrated through a willingness to discuss a future plan (lines 45–49). Leaning on techniques to learn from participants as experts in their own lives while encouraging participants to choose a goal that feels important and doable to them, allows participants to utilize the oral health education in a way they find achievable in their daily lives. Briefly comparing this exchange between participant and CHR appears more involved, trusting, and open—unlike the formative assessment descriptions of patient interaction with providers (Figure 2).

In another example, a participant works through setting a goal of brushing/wiping out the baby's mouth twice a day. The participant has been very active in the session prior to the goal setting portion, and the CHR responds to that participant by encouraging confidence in making changes going forward:

The CHR works with the participant to help think through the goal they want to set, the achievability of the goal, and the long-term benefits. The CHR frequently encourages the participant's confidence by supporting the goal and its outcomes. In lines 3–4, the CHR echoes the participant's sentiment of “a battle she's willing to fight.” In lines 11–16, the participant clearly outlines several goals for herself and her baby, these include: wiping/brushing the baby's teeth 2 times a day, maybe more if the participant can wipe out the baby's mouth after each feeding, and she concludes by making her own goal of modeling brushing for her baby. The participant also acknowledges that at 3:30 am, it may be tough to wipe out the baby's mouth—realistically setting her goals. The CHR also reviews the goal the participant states in lines 17–19, noting the participant is energetic about setting a goal for herself and her child. In lines 21–22, the CHR summarizes the goal by going back over the session information with the participant, with the participant confirming in line 23 that she will use a circular motion when wiping the baby's mouth. As part of the goal change worksheet, the CHR queries the participant's confidence in lines 24–26. Reporting high confidence in making this change in line 27, the CHR asks 138-7 to explain her confidence by putting it into words. The CHR reflects on the participant's opening statement of a “battle she's willing to fight” in line 28 and the participant confirms again, in line 29. And in lines 32–37, the participant reflects on her own characteristics, experiences, and family knowledge, which will help her achieve her goal. In addition, she comments on the real-world implications of these health choices and their cost—going into the complications of insurance and out of pocket costs, which are influential in motivating the participant to take action at home. Two weeks post-intervention, the same participant reported:

**NAU Research Team** [00:05:00] So you just told me a ton of information that you learned [in your session], which is amazing, but did any one of those things… have a real impact on you?

**138-7** [00:05:22] Yeah, it did, because like I said, I didn't know that I could've been brushing her gums prior to her teeth. And she had, she had just got like her teeth were just breaking through when we met with [CHR]. So like I had already bought, oh, the little like pens like, baby toothbrush and kind of one just to rub her teeth and stuff. But soon as she said that we started right away and literally we made it a goal. Like I started once a day brushing her teeth, her gums and like the teeth that was starting to break out. And now we're up to two [a day]. It got us to start brushing her gums and teeth right away.

The goal setting discussion with CHR turned into a new practice for participant 138-7. By supporting participant 138-7, working through her concerns and educating her on proper wiping and brushing of her child's mouth, CHR helped set up a successful at home behavior change plan.

## Discussion

This study demonstrated that the involvement of local CHRs who were able to create trusting relationships with participants, utilizing adapted MI and culturally tailored oral health education, addressed a need in the partnered communities. Formative assessment data showed a communication and education gap with dental patients. GBHNS was able to utilize local community members (CHRs) to lead consistent culturally tailored oral health education sessions with participants through adapted MI, resulting in more conversational education and satisfaction (exhibited by Transcript 1 and 2, Table 2).

Oral health provider reports of patient interactions outlined unproductive encounters. Patients did not appear concerned about decay according to providers and providers expressed uncertainty of how to reach patients. To help in these situations, providers wanted programs relying on local “cultural experts” to assist with high-risk patients. Using local CHRs, GBHNS was able to meet participants in a comfortable setting to conversationally complete an oral health education session. Transcripts 1 and 2 illustrate active participants, the result of built rapport as they work through how to apply oral health education to their own practices at home.

GBHNS also utilized local community advisory boards (CABs) to inform the intervention, local imagery and art as part of the education materials (Kirby et al., in review), along with local CHRs to deliver the intervention. Combining so many layers of familiarity into the intervention, GBHNS created a community centered intervention that seemed to resonate with participants. Building rapport is a well-known qualitative interviewing technique. In the GBHNS program, CHRs were viewed as local and familiar to participants by being part of the same community and members of the tribe. CHRs trained in research methods and adapted MI are able to uncover information from participants in a way non-community members may not. This is specifically striking in comparison to the formative assessment data which revealed communication problems between providers and patients.

Studies across disciplines find immense value in utilizing CHWs (or CHRs) to better engage the community, create mutual learning, and operate with sustainability in mind (29). For these programs to be impactful, Smith and Blumenthal urge community commitment to the project; CHWs must have the desire to bridge the research to practice gap, and training is fundamental to the project success (2012). Community commitment is described in Camplain et al. (6) and a forthcoming manuscript (Baldwin et al., in review). Not only did the community support help encourage women to participate in this study, but left participants inquiring with CHRs about engaging in additional education sessions. The clear lack of oral health education communication between dental providers and patients was a central motivator for CHRs to create engaging educational materials for their communities. By delivering this information to fellow community members, CHRs were able to engage in trusting conversations about at-home behaviors and generate individualized change plans for each participant. This resulted in all but one participant reporting a changed at-home oral health behavior post intervention.

## Limitations, strengths, and future work

One of the limitations of this study is the self-reported data. While participants reported at-home oral health behavior changes in their debriefing interviews, there was no additional verification of this report. In addition, while CHRs are part of communities and that involves familiarity and trust, some participants might have been concerned about confidentiality issues in small communities.

Despite these limitations, there were many strengths to this study. In addition to the positive impact of the intervention sessions, valuable educational materials were created that will have a lasting impact for each community. Specifically, more than 15 flip charts have been distributed to both communities. One community's sponsored CHR program has adopted the GBNHS oral health education program in coordination with the dental program for the 2025 year. Post intervention, the GBHNS team met with available providers who participated in the formative assessment at each site. Presentations of findings were made to dental providers and their teams. These presentations were met with excitement and engagement. At each provider's practice or office, GBHNS left the oral health education materials for providers to use or leave in their waiting rooms for patients to utilize.

In future work, it is of note that 7 of the 27 debriefing interviews revealed participants had brought children, partners, and/or parents with them to the oral health session. Feedback also allowed the GBHNS team to consider edits to the educational materials so that it was possible to involve more of the family in future interventions. Participants discussed living with other family members and how those individuals were involved in caring for their children. Reflecting this reality of family and intergenerational caretaking in the educational materials is another way of building rapport with the community and expanding oral health education.

## Conclusion

This study emphasizes the critical need for culturally tailored oral health education and communication within Indigenous communities. Formative assessment interviews revealed community level issues with oral health communication, including inconsistent oral health education, lack of trust in dental providers, and ongoing service disruptions. Caregivers often relied on non-dental sources for oral health information—particularly community-based programs and intergenerational family knowledge. Oral health providers expressed frustration with the limited reach and effectiveness of their efforts.

The implementation of the culturally tailored oral health intervention, delivered by trusted CHRs using adapted MI techniques, addressed many of the challenges identified by both caregivers and providers in the formative assessment. Participants reported high satisfaction with the sessions and also reported short-term changes in at-home oral health behaviors, including increased brushing, reduced consumption of sugary foods and beverages, and enhanced engagement in their children's oral health routines. Specifically, the goal-setting discussions in the intervention session fostered collaborative problem-solving and supported participant autonomy.

This intervention cultivated trust between CHRs and participants, creating an interpersonal dynamic not commonly reported in dental settings according to formative assessment data. The community-based relationship and flexible sessions allowed CHRs to tailor their communication to the needs and lived experience of each caregiver. This is in direct contrast to what was discovered in the formative assessment data, which emphasized communication breakdowns and unmet needs. The CHR based intervention sessions demonstrated how culturally relevant and conversational engagement can motivate oral health behaviors and education. Other studies focused on trust building with CHWs have claimed a need for illustrating how trust is built into community health work and here we present two specific examples from a culturally tailored oral health intervention.

Future efforts should focus on sustainability and feasibility of this culturally tailored approach. Training additional CHRs in adapted MI and ensuring continuity of local education programming could significantly improve oral health equity for Indigenous children and families. This intervention provides a replicable model that leverages community trust and culturally informed communication to bridge community identified gaps in oral health education services.

## Data Availability

The original contributions presented in the study are included in the article/Supplementary Material, further inquiries can be directed to the corresponding author.
